# Misdirected central venous catheter

**DOI:** 10.4103/0974-2700.62100

**Published:** 2010

**Authors:** Nita D'souza, Babita Gupta, Chhavi Sawhney, Anurag Chaturvedi

**Affiliations:** Department of Anesthesia and Intensive Care, Jai Prakash Narayan Apex Trauma Center, All India Institute of Medical sciences, New Delhi-110 029, India

Sir,

Central venous catheter (CVC) placement is a routine procedure in the management of critically ill patients. However, the placement of these catheters is not without risk. In the standard technique central venous cannulation is a blind procedure, which relies on the use of external anatomical landmarks. Correct placement of the CVC is an essential prerequisite for accurate monitoring of central venous pressure (CVP) and long-term use of the catheter. The complications of central venous cannulation are numerous and include malpositioning, a known complication with the reported incidence ranging widely from less than 1% to more than 60%.[1]

**Figure 1:**
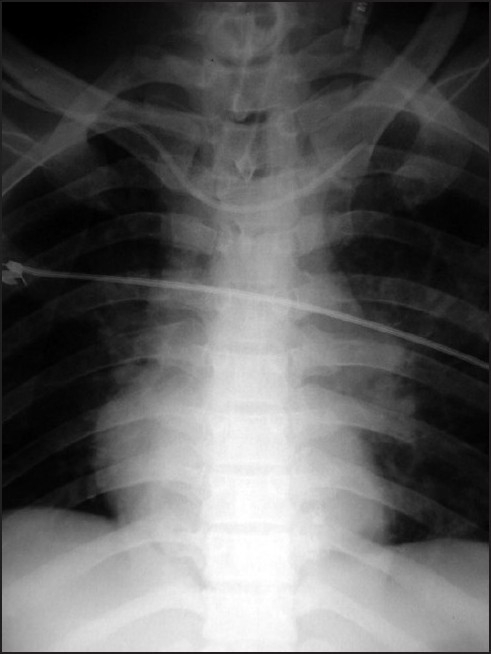
Central venous catheter malpositioned in the contralateral subclavian vein

We present a case where the CVC was malpositioned in the contralateral subclavian vein. Right subclavian central venous access was performed in a 34-year-old critically ill tracheostomized patient in early sepsis with a 7 French triple-lumen catheter (BBraun) and fixed at the 16-cm mark at the skin level. On connecting the transducer to the monitor there was normal CVP waveform and free aspiration of venous blood. However, the chest roentgenogram revealed the tip of the central line to be seated in the contralateral side, i.e., in the left subclavian vein [Figure 1]. This malposition was unexpected as the pressure waveform was normal. The catheter was withdrawn 1½ cm and was subsequently radiographically confirmed to be in correct position.

Malpositioning of catheter tip in contralateral subclavian vein is an extremely unusual occurrence, although the incidence has not been stated clearly in studies. Other unusual catheter tip placements reported are in the left internal mammary vein, azygous vein, hemiazygos vein, lateral thoracic vein, inferior thyroid vein, left superior intercostal vein, thymic vein, pleural cavity, and the jugular foramen.[2–4] During subclavian vein catheterization, the most common misplacement of the catheter is cephalad, into the ipsilateral internal jugular vein (IJV).

After any repositioning of the CVC, chest radiography is required for confirmation. One study reported that manual occlusion of the ipsilateral IJV during subclavian vein cannulation reduced the chances of advancement of the CVC into the ipsilateral IJV. The IJV occlusion test (applying external pressure on the IJV for approximately 10 seconds in the supraclavicular area and observing the changes in the CVP and its waveform pattern) successfully detects misplacement of a subclavian vein catheter into the IJV. However, it does not detect any other misplacement.[3] Some authors implicate excessive lengths of guidewire as the cause.[4] Other authors consider the length of the CVC inserted itself to be a risk factor. Studies have hypothesized that the final position of the catheter tip depends on the course that the guidewire takes. This may be influenced by the initial orientations of the J-type guidewire tip during the subclavian approach.[5] We currently perform cannulations keeping the J-tip of the guidewire directed caudad and have found that this increases correct placement of CVC in the right atrium.

A 29% incidence of malposition was reported by Deitel and McIntyre in post-cannulation chest radiographs.[6] Misplacement of the catheter tip can enhance the risk of clot formation and cause thrombophlebitis and catheter erosion, apart from impairing CVP measurement. Moreover, malposition of a CVC in a small-caliber vessel can result in inaccurate readings and is also undesirable for hyperalimentation and other situations requiring long-term infusions.[7] Reinsertion of the catheters is not without potential complication and there is also the possibility of repeating the malposition.

Although radioimaging is the gold standard for confirming the correct positioning of the CVC, there may be a delay. The use of ultrasound (USG) to guide insertion of CVC is controversial. Some authors suggest that USG guidance improves the success rate of subclavian venous catheterization performed by relatively less experienced operators. On the other hand, others have reported that USG guidance had no effect on the rate of complications or failures of subclavian vein catheterization.[8] Hence, insertion of CVC remains essentially a blind procedure that is guided by anatomical landmarks.

We write this letter to reiterate the importance of careful radiological imaging as early as possible to verify correct placement of a CVC. The catheter tip could produce ideal central venous waveforms despite being seated at an undesirable site.
